# Agreement of imageless navigation‐derived pelvic tilt measurements with radiographic and CT‐based measurements in direct anterior total hip arthroplasty: A prospective single‐center study

**DOI:** 10.1002/jeo2.70842

**Published:** 2026-07-07

**Authors:** Roham Borazjani, Danielle DeMoes, Tristan Jones, Carmelo Burgio, Anthony Gualtieri, Jonathan Vigdorchik, Stefan Kreuzer

**Affiliations:** ^1^ INOV8 Research Houston Texas USA; ^2^ Naviswiss Brugg Switzerland; ^3^ Adult Reconstruction and Joint Replacement Service, Department of Orthopaedic Surgery Hospital for Special Surgery New York New York USA; ^4^ INOV8 Healthcare Houston Texas USA

**Keywords:** agreement analysis, direct anterior approach, imageless navigation, pelvic tilt, total hip arthroplasty

## Abstract

**Purpose:**

Pelvic tilt (PT) is a major determinant of functional acetabular orientation, affecting implant stability and impingement risk. Although imageless navigation systems are increasingly used to guide intraoperative cup orientation, their validity in measuring supine PT remains unclear. This study evaluated the validity of an imageless navigation system relative to preoperative computed tomography (CT)‐based planning and intraoperative radiographic measurements during direct anterior approach (DAA) total hip arthroplasty (THA).

**Methods:**

This prospective observational study included consecutive patients undergoing primary DAA‐THA between August 2023 and June 2024 at a single ambulatory surgery center. Sagittal PT was measured intraoperatively using imageless navigation and compared with CT‐based preoperative planning and cross‐table intraoperative lateral X‐ray measurements. Agreement among the navigation system, lateral X‐ray and preoperative planning was assessed. Demographic characteristics, morphometric variables and suprapubic fat thickness (SPFT) were collected to assess their associations with measurement error.

**Results:**

Fifty‐one patients were included (64.7% female; mean age, 59.6 ± 10.8 years). Bland–Altman analysis showed greater bias for X‐ray_Navigation (3.7 ± 3.6°; limits of agreement [LoA]= −3.3° to 10.7°) and Navigation_CT (3.6 ± 4.9°; LoA = −6.0° to 13.3°) than for CT_X‐ray (−0.1 ± 3.6°; LoA = −7.2° to 7.0°). Mean absolute error (MAE) was 4.1 ± 3.1°, 2.9 ± 2.2° and 4.8 ± 3.8° for X‐ray_Navigation, CT_X‐ray, Navigation‐CT, respectively. SPFT independently predicted increased MAE in both the X‐ray_Navigation and Navigation_CT comparisons. Receiver operating characteristic analysis identified SPFT cutoffs of 48.9 and 47.1 mm for predicting ≥5° discrepancies in the X‐ray–Navigation and Navigation–CT comparisons, respectively.

**Conclusion:**

The imageless navigation device demonstrated moderate agreement with CT‐based and intraoperative radiographic measurements and may provide clinically useful intraoperative estimation of supine PT during DAA‐THA. However, SPFT was independently associated with larger measurement discrepancies.

**Level of Evidence:**

Level III.

**Abbreviations**
APP: anterior pelvic plane
ASC: ambulatory surgery center
BMI: body mass index
CI: confidence interval
CT: computed tomography
DAA: direct anterior approach
FDA: Food and Drug Administration
ICC: intraclass correlation coefficient
IQR: interquartile range
IRB: institutional review board
MAE: mean absolute error
OPS: Optimized Positioning System
OR: odds ratio
PT: pelvic tilt
SD: standard deviation
SJR: Surgical Joint Registry
SPFT: suprapubic fat thickness
STROBE: Strengthening the Reporting of Observational Studies in Epidemiology
THA: total hip arthroplasty

## INTRODUCTION

Optimal acetabular cup orientation during primary total hip arthroplasty (THA) is pivotal for preventing complications such as instability, dislocation, impingement, wear and aseptic loosening [13, 21, 22, 23]. Historically, an inclination of 40 ± 10° and anteversion of 15 ± 10° have been considered ‘safe’ for acetabular cup orientation [20]. However, these recommendations were derived from a small retrospective study, and recent literature has questioned the universality of these safe zones [1, 5, 8]. More recently, the concept of the functional safe zone has emerged, recognizing that acetabular orientation is not static but varies with changes in sagittal pelvic position, commonly referred to as pelvic tilt (PT), across functional activities [18].

The relationship between PT and acetabular cup orientation has been well studied. Posterior PT beyond neutral, resulting in an outlet pelvis, increases functional cup anteversion, whereas anterior PT (an inlet pelvis) decreases functional cup anteversion. Furthermore, for every 1° change in PT, functional acetabular anteversion changes by approximately 0.5°–1° [33]. Consequently, accurate evaluation and understanding of a patient's PT play a critical role in achieving optimal acetabular cup orientation during THA [8, 26].

Recent studies have emphasized the dynamic nature of the pelvis, with the magnitude and direction of pelvic motion varying among individuals [24, 30]. The pelvis rotates across different functional positions, with an average posterior pelvic rotation of approximately −5° from supine to standing (range: −21.8 to 8.4°), −4° from supine to flexed seated (range: −48.3 to 38.6°) and 2° from standing to flexed seated (range: −51.8 to 39.5°) [6]. This variability in functional pelvic rotation necessitates patient‐specific intraoperative customization to minimize the risk of postoperative functional malpositioning and dislocation [8].

Navigation systems have emerged as valuable tools for enhancing the precision of acetabular cup orientation, contributing to improving the accuracy of both inclination and anteversion [12, 19]. Portable navigation systems, comprising accelerometers, gyroscopes and inertial sensors, have become increasingly used in THAs [12, 17, 32]. The Naviswiss system (Naviswiss AG) is a Food and Drug Administration (FDA)‐approved handheld navigation device that enables intraoperative registration of anatomical landmarks, with or without preoperative computed tomography (CT) imaging [32]. The system features an infrared stereo camera that assesses the position and orientation of the pelvis in space [17]. When combined with functional preoperative imaging, this information may allow surgeons to target patient‐specific acetabular cup orientation intraoperatively. Notably, the navigation system is an open platform, allowing implant selection at the surgeon's discretion.

Despite growing recognition of the dynamic and patient‐specific nature of PT and its influence on acetabular orientation, limited evidence exists regarding the agreement of intraoperative PT measurements obtained using imageless navigation systems in the supine position during direct anterior approach (DAA) THA, compared with established methods such as preoperative CT‐based planning and intraoperative radiographic assessment. Therefore, this study aims to evaluate the validity and agreement of the intraoperative imageless navigation system in assessing sagittal pelvic position. We hypothesized that imageless navigation would demonstrate clinically acceptable agreement with CT‐ and radiograph‐based PT measurements.

## METHODS

### Study design and setting

This prospective observational study was conducted at our ambulatory surgery center (ASC) in Houston, Texas. All procedures were performed by the senior fellowship‐trained surgeon (S.K.) using DAA with the ARCH Leg Positioning System (IOT AG). All participants provided informed consent for inclusion in the registry. This study was conducted in accordance with the STROBE (Strengthening the Reporting of OBservational studies in Epidemiology) guideline [7] and complied with the principles of the Declaration of Helsinki and the subsequent amendments [9].

### Participant selection

**Figure 1:**
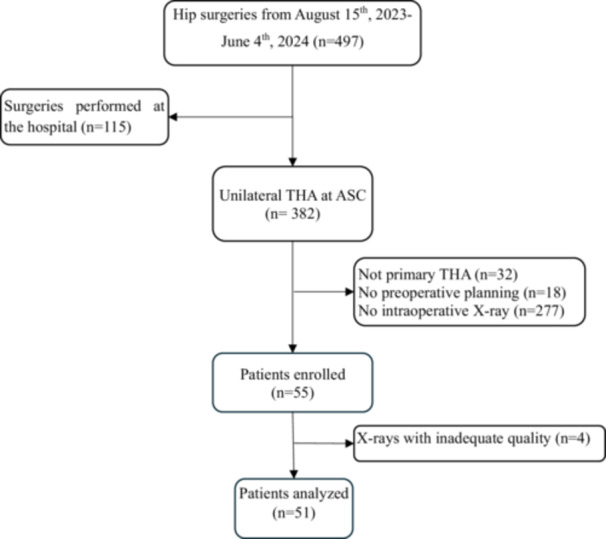
Patients' flowchart. ASC, ambulatory surgery center; THA, total hip arthroplasty.

All consecutive patients undergoing primary unilateral THA via a DAA for primary osteoarthritis using the *image‐based* version of the navigation system (Naviswiss AG) between August 2023 and June 2024 were screened for eligibility. Additional inclusion criteria were: (1) availability of a preoperative CT scan and (2) provision of informed consent for participation in the registry. Exclusion criteria were (1) revision or conversion THA, (2) lack of intraoperative cross‐table lateral X‐ray images and (3) incomplete radiographic data and/or structural or anatomical abnormalities affecting pelvic morphology (Figure 1). The senior surgeon had more than 15 years of experience using various navigation systems, with more than 5000 procedures performed using these systems; therefore, no learning curve was considered for this study. *Image‐based* navigation has been used at our ASC and remains part of routine surgical practice based on the senior surgeon's clinical preference, and its validity and accuracy have been previously investigated and approved [29]. The *imageless* system, along with intraoperative cross‐table lateral radiographs, was used investigatively and independently to obtain intraoperative PT. This study was not designed to compare image‐based and imageless navigation systems for cup placement accuracy, and cup positioning data from the image‐based system were not used to validate PT measurements.

### Data collection

Demographic and morphometric variables including age, sex and body mass index (BMI) were collected retrospectively from our institutional database (IRB: 20203266). Our database, the Surgical Joint Registry (SJR), is a prospective cohort that captures perioperative data for all patients who undergo joint replacement procedures performed by our surgeons. Adverse events were defined as any revision, dislocation or rehospitalization after the index surgery and were identified from the index procedure through the last available follow‐up visit using our database. Any missing values were captured through medical record review.

### Data sources and measurement

Preoperative CT scans were obtained for surgical planning and measurement of native PT according to the Optimized Positioning System (OPS; Corin Group) protocol in all patients. CT‐based measurements were performed following the methodology described by Pierrepont et al. [26]. Suprapubic fat thickness (SPFT) was retrospectively measured on axial CT images as the shortest perpendicular distance from the anterior cortex at the superior margin of the pubic symphysis to the overlying skin surface at the midline [14].

**Figure 2:**
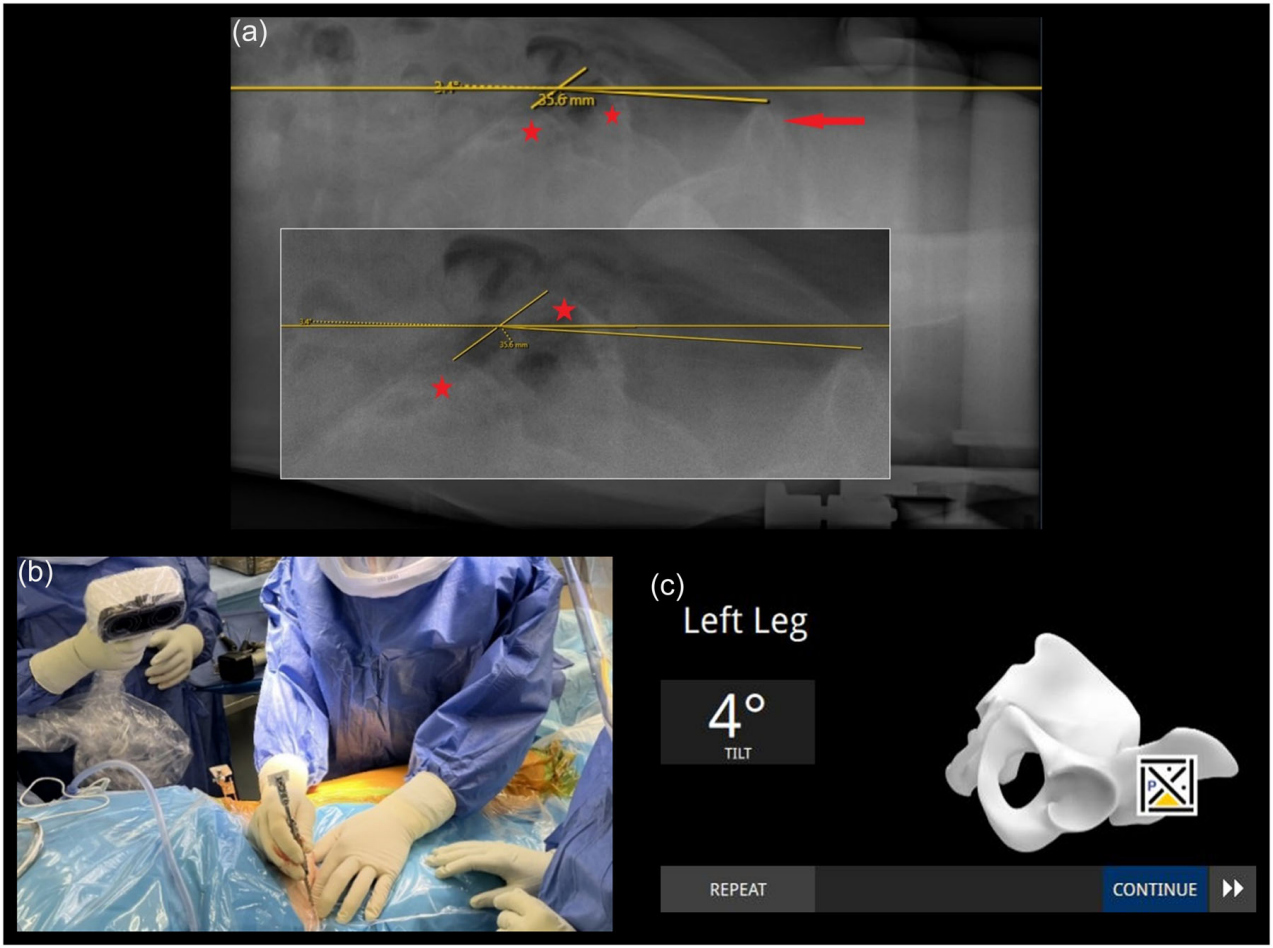
Pelvic tilt measurement methods. (a) PT measurement on lateral X‐ray using the pubic symphysis and bilateral ASIS landmarks. (b, c) Registration of the same landmarks using the imageless navigation system. ASIS, anterior superior iliac spines; PT, pelvic tilt.

A standardized intraoperative cross‐table lateral radiograph was obtained immediately before navigation registration, and radiographic PT was measured using the same anterior pelvic plane (APP) reference. This intraoperative radiographic measurement served as the reference standard for the primary comparison with imageless navigation because it was obtained under the same anesthetized operative conditions as the navigation measurement (Figure 2a). In contrast, CT‐based PT represented the preoperative value; therefore, disagreement between CT and intraoperative measurements could not be interpreted solely as measurement error, as it may also reflect true positional or physiologic changes in PT related to anesthesia, patient movement and surgical positioning. Sagittal PT was defined as the angle between the APP, formed by the bilateral anterior superior iliac spines and the pubic symphysis and the horizontal line as shown in Figure 2 [15, 16].

The imageless navigation workflow involved intraoperative identification and registration of the APP using a blunt pointer according to standard surgical technique. A built‐in inertial measurement unit quantified APP orientation in the supine position relative to the horizontal plane (Figure 2b,c). Each patient underwent two intraoperative navigation registration cycles, and measurements were considered acceptable if the difference between cycles was within 5° according to the manufacturer‐recommended registration protocol. After confirming PT measurements with the *imageless* platform, the surgery was resumed using the *image‐based* navigation platform as part of the standard workflow. The validity and accuracy of this image‐based navigation system have been reported previously [29].

To minimize measurement bias, all intraoperative radiographs were obtained using a standardized technique. Radiographic PT measurements were independently performed by two observers (R.B. and T.J.), both blinded to each other's assessments and to the CT‐ and navigation‐derived measurements. Each observer repeated the measurements twice, at least one month apart, using an FDA‐approved software platform (eVue; Efferent Healthcare). The average of the four measurements was used as the final X‐ray–based PT value. Navigation‐derived measurements were recorded automatically without manual input. Intraobserver reliability for PT measurements was excellent (intraclass correlation coefficient [ICC] = 0.99 and 0.98, respectively), and interobserver reliability was also high (ICC = 0.9; 95% confidence interval [CI]: 0.8–1.0; *F*(51) = 25.5; *p* < 0.001).

### Data analysis

The primary outcome was agreement between supine PT measurements obtained using the imageless navigation platform and intraoperative cross‐table lateral X‐ray measurements, which were used as the reference values. Secondary outcomes included agreement between imageless navigation and CT‐based preoperative planning measurements, discrepancies in PT measurements between platforms and associations between morphometric variables and these discrepancies.

Continuous variables were summarized using mean ± standard deviation (SD) or median with interquartile range (IQR), as appropriate, and categorical variables were reported as frequencies and percentages. Agreement among measurement methods was assessed using Bland–Altman analysis, with mean bias and 95% limits of agreement (LoA) reported for each comparison. Mean absolute error (MAE) between the three modalities was compared using the Kruskal–Wallis test, followed by pairwise Wilcoxon rank‐sum tests with Holm adjustment for multiple comparisons. Associations between morphometric and demographic variables (age, sex, BMI and SPFT) and MAE were assessed using Pearson correlation, Wilcoxon rank‐sum test or Kruskal–Wallis test. Multivariable linear regression analyses were then performed to identify independent predictors of MAE. Receiver operating characteristic (ROC) curve analysis was used to evaluate the ability of the predictor to identify clinically meaningful PT discrepancies ≥5° and to determine optimal cutoff values. Post hoc power analysis demonstrated sufficient power (75.0%, *α* = 0.05) to detect a mean MAE below the predefined clinical acceptability threshold of 5°. Importantly, despite this moderate power, the sample size allowed for precise estimation of measurement error, with the mean MAE estimated within approximately ±1° at the 95% confidence level, supporting clinical interpretability. A two‐sided *p* value < 0.05 was considered statistically significant. All analyses and figure generation were performed using RStudio (R Foundation for Statistical Computing).

## RESULTS

A total of 51 patients (64.7% female) met the inclusion criteria, with a mean age of 59.6 ± 10.8 years (range: 27–84 years). The median intraoperative supine PT measured using the imageless navigation system was 2.6° (IQR, −1.8° to 8.5°), compared with 6.0° (IQR, 4.0°–9.0°) on CT images and 6.8° (IQR, 2.2°–10.4°) on intraoperative lateral X‐ray. Detailed demographic characteristics are provided in Table 1 and Table S1.

**Table 1:** Patient demographics.

Variables	
Age (years)^a^	59.6 ± 10.8
Gender, *n* (%)	
Male	18 (35.3)
Female	33 (64.7)
BMI (kg/m^2^)^a^	28.2 ± 4.8
BMI categorical (kg/m^2^), *n* (%)	
BMI < 25.0	16 (31.4)
25.0 ≤ BMI < 30.0	17 (33.3)
BMI ≥ 30.0	18 (35.3)
Suprapubic thickness (mm)	47.3 ± 14.8
Pelvic tilt measurement, median (IQR)	
Imageless navigation system	2.6° (−1.8° to 8.5°)
Computed tomography	6° (4°–9°)
X‐ray	6.8° (2.2°–10.4°)

Abbreviations: BMI, body mass index; IQR, interquartile range; mm, millimeter.

^a^ Mean and standard deviation.

**Figure 3:**
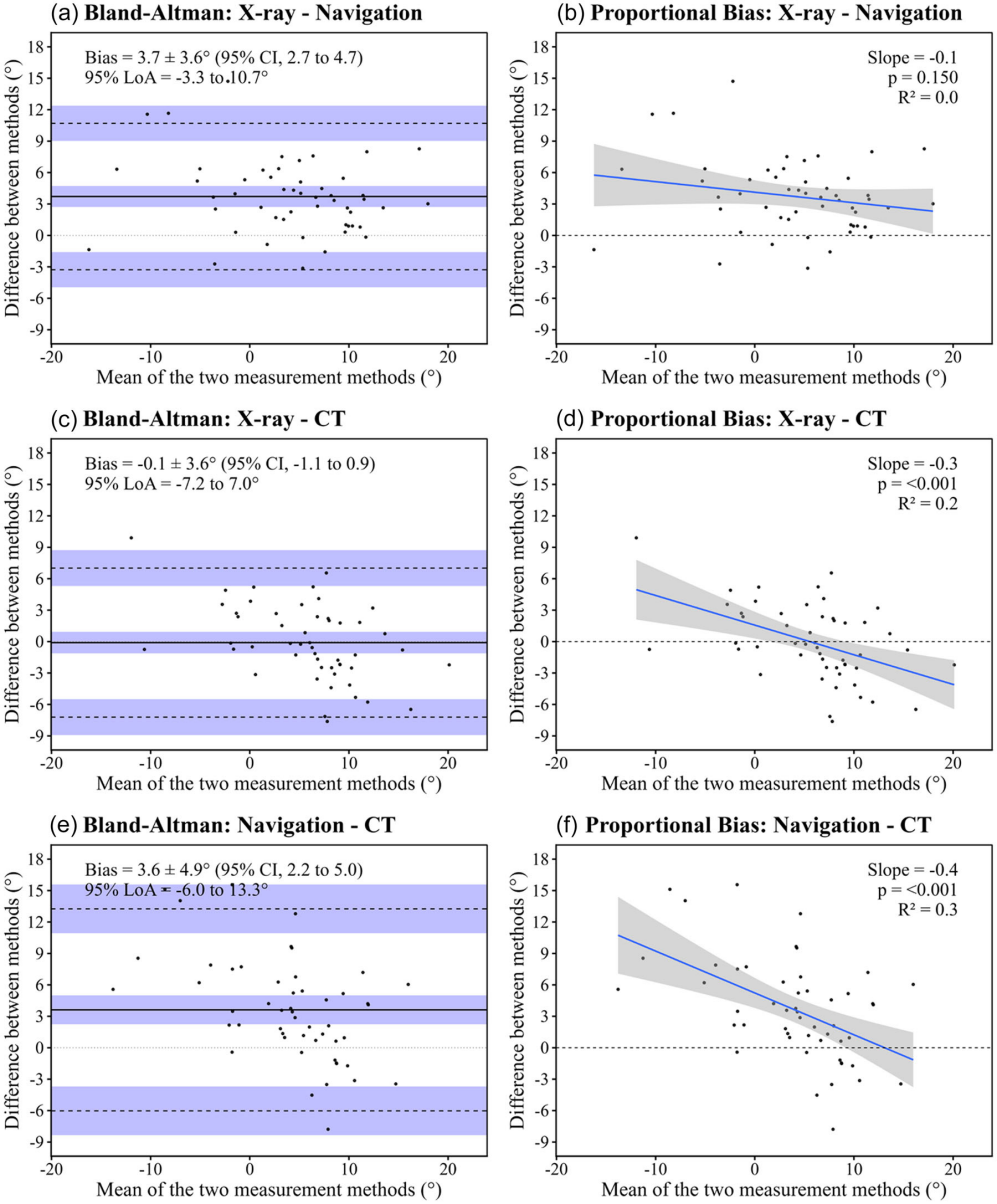
Bland–Altman and propotional bias analyses reporting the agreement in supine pelvic tilt measurements between X‐ray versus navigation (a, b), X‐ray versus CT (c, d), and navigation versus CT (e, f). CI, confidence interval; CT, computed tomography; LoA, limits of agreement.

Bland–Altman analysis demonstrated a mean difference of 3.7 ± 3.6° between X‐ray and navigation measurements, with 95% LoA ranging from −3.3° to 10.7° (Figure 3a). Proportional bias analysis showed no statistically significant bias (slope = −0.1, *p* = 0.150 and *R* ^2^ = 0.04, Figure 3b). When X‐ray was compared with CT, the mean difference was −0.1 ± 3.6° (95% LoA = −7.2° to 7.0°), indicating minimal average bias relative to CT (Figure 3c). However, proportional bias was observed for X‐ray versus CT, with a slope of −0.3 (*R* ^2 ^= 0.2, *p* < 0.001, Figure 3d). Navigation demonstrated a mean difference of 3.6 ± 4.9° compared with CT, with wider 95% LoA ranging from −6.0 to 13.3° (Figure 3e). Proportional bias was also significant for navigation versus CT (slope = −0.4, *R* ^2 ^= 0.3, *p* < 0.001), indicating that the magnitude of disagreement between navigation and CT varied across the range of PT values (Figure 3f).

**Figure 4:**
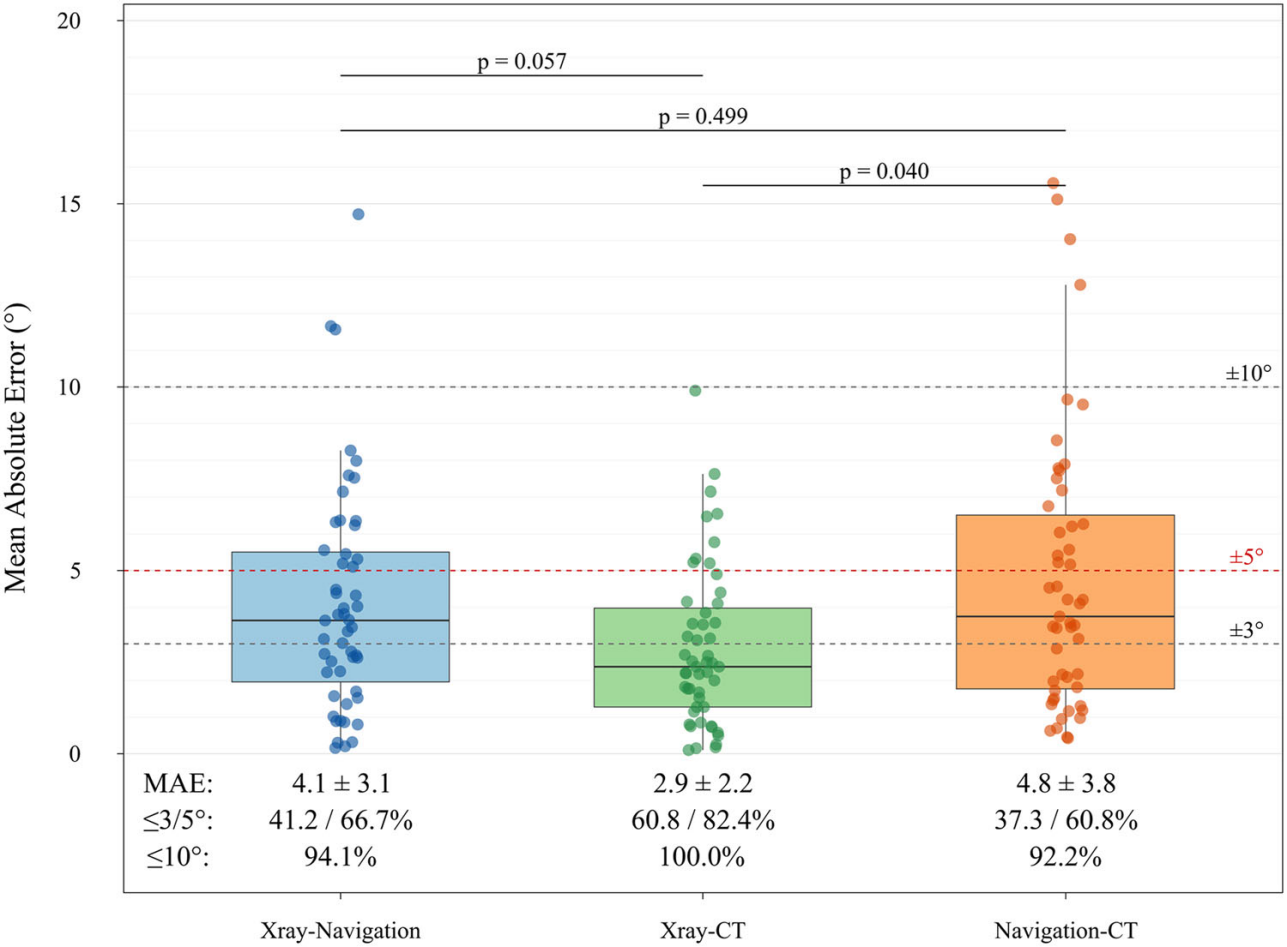
Boxplot showing MAE between these measurement methods. Non‐parametric analyses were used because the variables were not normally distributed (X‐ray_Navigation: *p* < 0.001; X‐ray_CT: *p* = 0.002; Navigation_CT: *p* < 0.001). CT, computed tomography; MAE, mean absolute error; SD, standard deviation.

The corresponding MAE values for X‐ray_Navigation, Navigation_CT and CT_X‐ray were 4.1 ± 3.1° (95% CI: 3.2°–5.0°), 4.8 ± 3.8° (95% CI: 3.7°–5.8°) and 2.9 ± 2.2° (95% CI: 2.3°–3.5°), respectively (Figure 4). The proportion of PT error within 10° was higher for the X‐ray_Navigation comparison (94.1%) than for the Navigation_CT comparison (92.2%). Even using a more stringent 5° cutoff, a higher proportion of measurements fell within this threshold for X‐ray_Navigation than for Navigation_CT (66.7 vs. 60.8%).

**Figure 5:**
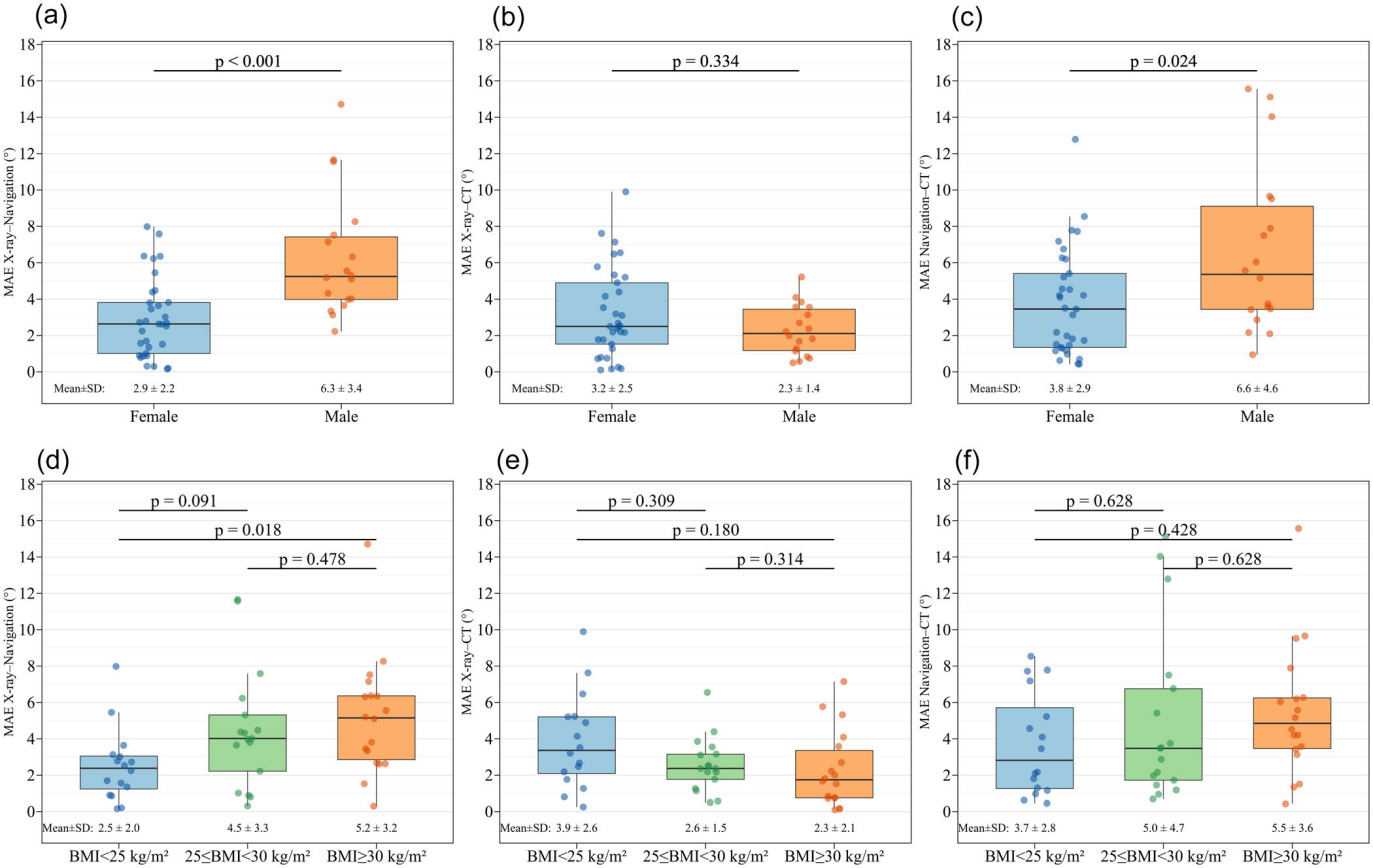
Comparison of mean absolute error by sex and body mass index: (a) X‐ray versus navigation by sex; (b) X‐ray versus CT by sex; (c) navigation versus CT by sex; (d) X‐ray versus navigation by BMI category; (e) X‐ray versus CT by BMI category; and (f) navigation versus CT by BMI category. BMI, body mass index; CT, computed tomography.

PT measurement error differed significantly by sex. Male patients had higher MAE than female patients in both the X‐ray_Navigation comparison (6.3 ± 3.4° vs. 2.9 ± 2.2°; *p* < 0.001; Figure 5a) and the Navigation_CT comparison (6.6 ± 4.6° vs. 3.8 ± 2.9°; *p* = 0.024; Figure 5c). When BMI was analyzed categorically, MAE in the X‐ray_Navigation comparison also differed significantly across BMI groups, with higher error observed in patients with obesity (BMI ≥ 30 kg/m^2^; 5.2 ± 3.2°) compared with those with normal BMI (<25 kg/m^2^, 2.5 ± 2.0°, *p* = 0.018; Figure 5d).

**Figure 6:**
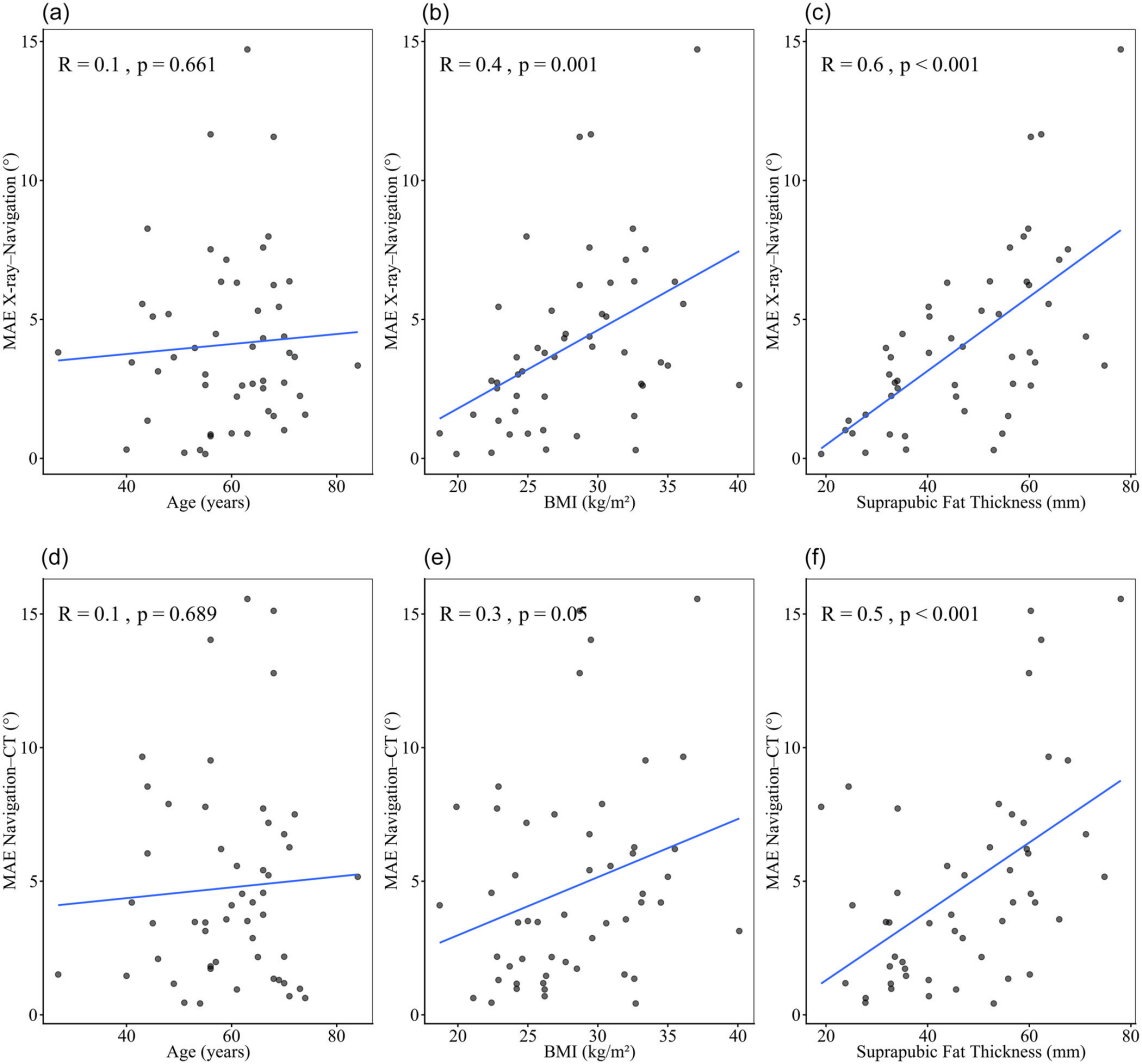
Pearson correlation between mean absolute error and anthropometric variables: (a) X‐ray versus navigation error and age; (b) X‐ray versus navigation error and BMI; (c) X‐ray versus navigation error and suprapubic fat thickness; (d) navigation versus CT error and age; (e) navigation versus CT error and BMI; and (f) navigation versus CT error and suprapubic fat thickness. BMI, body mass index; CT, computed tomography; MAE, mean absolute error.

Further morphometric analyses demonstrated no significant association between age and MAE in either comparison (*p* > 0.05; Figure 6a,d). In contrast, BMI showed a significant positive correlation with MAE in the X‐ray_Navigation comparison (*R* = 0.4, *p* = 0.001) and Navigation_CT comparison (*R* = 0.3, *p* = 0.05, Figure 6b,e). SPFT showed the strongest morphometric association, demonstrating moderate positive correlations with MAE in both the X‐ray_Navigation (*R* = 0.6, *p* < 0.001) and Navigation_CT comparisons (*R* = 0.5, *p* < 0.001; Figure 6c,f).

In multivariable linear regression analyses, male sex was independently associated with increased MAE in the X‐ray_Navigation comparison (*β* = 2.3°, 95% CI: 0.7–3.8; *p* = 0.005), but not in the Navigation‐CT comparison (*β *= 1.8°, 95% CI: −0.4 to 3.9; *p* = 0.109). Age and BMI were not independently associated with MAE in either model. However, SPFT remained a significant independent predictor of increased MAE in both the X‐ray_Navigation (*β *= 0.1° per mm, 95% CI: 0.1–0.2; *p* = 0.002) and Navigation‐CT measurements (*β *= 0.2° per mm, 95% CI: 0.1–0.3; *p* = 0.004), indicating that greater anterior soft tissue thickness was consistently associated with increased measurement error (Table 2).

**Table 2:** Multivariable linear regression analyses for factors associated with measurement error.

Predictors	MAE X‐ray_Navigation		MAE Navigation_CT	
	*β* (95% CI)	*p* value	*β* (95% CI)	*p* value
Gender (male)	2.3 (0.7–3.8)	0.005*	1.8 (−0.4 to 3.9)	0.109
Age (years)	0.003 (−0.1 to 0.1)	0.931	−0.02 (−0.1 to 0.1)	0.730
BMI (kg/m^2^)	−0.1 (−0.3 to 0.2)	0.551	−0.2 (−0.5 to 0.1)	0.158
SPFT (mm)	0.1 (0.1–0.2)	0.002*	0.2 (0.1–0.3)	0.004*

Abbreviations: BMI, body mass index; CI, confidence interval; CT, computed tomography; MAE, mean absolute error; SPFT, suprapubic fat thickness.

* Significant *p* value.

**Figure 7:**
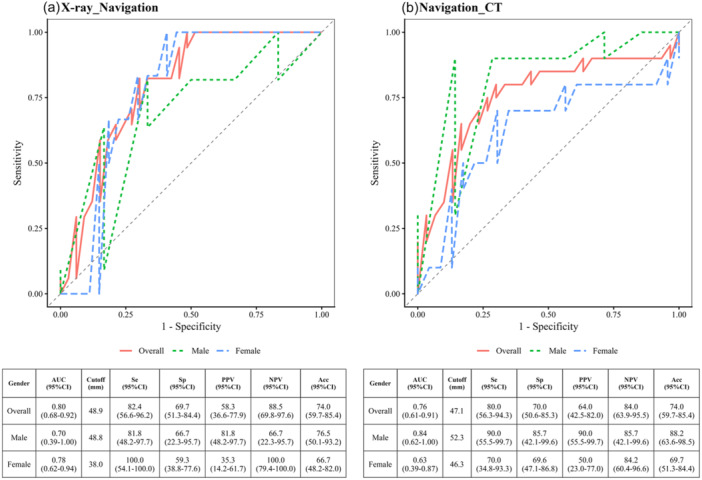
ROC analysis of SPFT for predicting pelvic tilt discrepancies ≥5°. ROC curves are shown for discrepancies between (a) intraoperative X‐ray and navigation and (b) navigation and preoperative CT. Acc, accuracy; AUC, area under the curve; CI, confidence interval; CT, computed tomography; NPV, negative predictive value; PPV, positive predictive value; ROC, receiver operating characteristic; Se, sensitivity; Sp, specificity; SPFT, suprapubic fat thickness.

ROC curve analysis demonstrated that SPFT had good overall discriminatory ability for predicting clinically meaningful PT discrepancies ≥5°. For the X‐ray–Navigation discrepancy, the overall area under the curve (AUC) was 0.8 (95% CI = 0.7–0.9), with an optimal SPFT cutoff of 48.9 mm, yielding 82.4% sensitivity and 69.7% specificity. When stratified by sex, the AUC was 0.7 (95% CI = 0.4–1.0) in males, with a cutoff of 48.8 mm, sensitivity of 81.8% and specificity of 66.7%; in females, the AUC was 0.8 (95% CI = 0.6–0.9), with a cutoff of 38.0 mm, sensitivity of 100.0% and specificity of 59.3% (Figure 7a). For Navigation_CT discrepancy, the overall AUC was 0.80 (95% CI = 0.6–0.9), with an optimal cutoff of 47.1 mm, sensitivity of 80.0% and specificity of 70.0% (Figure 7b).

At a mean follow‐up of 12.6 ± 7.8 months, no adverse events, including revision surgery, dislocation or rehospitalization, were observed. One patient underwent a second surgery for hardware removal due to cerclage‐induced iliopsoas tendinitis and pain 20 months after the index surgery. The cerclage wire had been placed during the index surgery to stabilize a small intraoperative periprosthetic fracture.

## DISCUSSION

In this study, we evaluated the agreement between imageless‐derived intraoperative PT measurements and PT measured using intraoperative lateral radiographs and preoperative CT planning. Overall, imageless navigation demonstrated moderate agreement with both comparators, with closer agreement observed relative to intraoperative radiographs than to preoperative CT. Additionally, the majority of PT measurements fell within accepted error thresholds (66.7% and 60.8% fell within 5° of MAE compared with X‐ray and CT images, respectively). Last but not least, increased SPFT was the most consistent independent predictor of increased error.

In our study, intraoperative navigation measurements consistently demonstrated less anterior PT than preoperative CT, with differences reaching up to 14° in some cases. In general, PT during primary THA is likely influenced by multiple factors such as patient positioning on a traction table, the use of muscle relaxation, limb manipulation and table adjustments, all of which differ from the static conditions under which preoperative supine CT imaging is obtained [27]. However, in the present study, positional variability was further reduced by taking intraoperative lateral x‐ray and consistently measuring PT in the supine position across modalities. These findings underscore the importance of real‐time intraoperative PT assessment using navigation, as reliance solely on preoperative imaging may not fully account for dynamic changes occurring during DAA THA [12, 17, 32].

Another potential reason for the observed differences in supine PT may be methodological variation between navigation‐based measurements and radiographic measurements obtained from X‐ray and preoperative CT. The navigation system uses a pointer to identify the pubic symphysis as an anatomical landmark, whereas X‐ray and CT rely on the radiographic pubic symphysis and therefore do not account for soft‐tissue thickness or other morphometric factors. However, according to the study conducted by Lembeck et al. [19], a PT discrepancy of this magnitude (4.8 ± 3.8°) would correspond approximately to a 3° change in functional cup anteversion and a 1.5° change in functional cup inclination, which is clinically negligible.

Measurement error was not uniform across patients and appeared to be influenced by morphometric factors. Although BMI showed moderate correlations with measurement error in univariable analyses, it was not independently associated with measurement error after multivariable adjustment. To the best of our knowledge, although several reports have addressed the effect of BMI on patients' outcomes [3, 4, 34], few studies have directly evaluated the effect of morphometric variables on PT, and almost all have focused on cup orientation accuracy, resulting in heterogeneous findings. Using a similar imageless navigation system, Ong et al. reviewed a cohort of 325 patients and reported that imageless navigation accurately measured cup orientation regardless of BMI [25]. Hohmann et al. evaluated the accuracy of the Stryker imageless navigation system (Stryker Corporation) and reported high accuracy in both patients with obesity and those without obesity [14]. In a separate prospective cohort using the same navigation system used in our study, Scholes et al. found that BMI was not associated with variation in cup measurements after anterolateral primary THA [28].

In contrast, SPFT emerged as the strongest independent predictor of increased measurement error across navigation‐based comparisons, suggesting that anterior soft tissue thickness interferes with reliable registration of pelvic landmarks, particularly the pubic symphysis, which serves as a critical reference point for imageless navigation systems. While BMI did not independently predict measurement error in the multivariable models, this finding underscores the importance of localized soft tissue distribution rather than global body habitus in determining the reliability of imageless navigation‐derived PT measurements. Based on ROC analysis, an SPFT of approximately 47 mm or greater was associated with an increased risk of clinically meaningful PT measurement error. Therefore, we suggest that if CT scans were performed preoperatively, surgeons could use this cutoff value to screen patients who may be at increased risk for imageless navigation THA. Therefore, preoperative CT‐based SPFT measurement may serve as a practical screening tool to identify patients in whom imageless navigation‐derived PT values should be interpreted with caution. In such patients, image‐based navigation may be considered. However, given the relatively small cohort size and the wide CIs observed in the sex‐stratified ROC analyses, these thresholds should be interpreted as exploratory rather than definitive. Future studies with larger cohorts are needed to validate these findings.

These results are partially consistent with prior studies evaluating the impact of soft tissue thickness on navigation‐guided acetabular component placement. Consistent with our findings, Hasart et al. demonstrated that symphyseal soft tissue thickness adversely affected the accuracy of ultrasound‐ and pointer‐based navigation systems, highlighting the sensitivity of landmark‐based registration to overlying soft tissue [11]. More recently, Suzuki et al. showed that both pubic symphysis and ASIS soft tissue thickness measured on preoperative CT were significant risk factors for acetabular cup malposition using a mechanical navigation device, whereas BMI alone showed a more limited association [31]. In contrast, Hohmann et al. reported no clinically meaningful association between soft tissue thickness measured over pubic tubercles and acetabular cup orientation using an imageless navigation system, although small inverse correlations were observed at the ASIS and were deemed nonsignificant due to low effect sizes [14]. This emphasizes that patient‐specific anterior pelvic anatomy, rather than overall BMI, should be carefully considered when interpreting navigation data during imageless, landmark‐based acetabular component orientation.

To our knowledge, this is among the first studies to assess the PT measurement agreement using a handheld imageless navigation system while incorporating detailed morphometric analyses. The observed moderate agreement between navigation measurements, preoperative planning and intraoperative X‐ray supports the validity of imageless navigation as a real‐time intraoperative tool. By identifying patient‐specific factors associated with increased error, this study provides insights that may improve interpretation of navigation data. However, our study was not without limitations. Because the study cohort was derived from a single ASC, involved a single surgeon with extensive experience in navigation‐assisted THA, and included only procedures performed through the DAA, the generalizability of the findings may be limited. Moreover, X‐ray‐ and navigation‐derived PT measurements were limited to the supine position and may not reflect functional pelvic orientation during standing or sitting. In addition, the mean follow‐up duration of 12.6 months was limited and did not meet the 30‐month minimum proposed for short‐term orthopedic follow‐up [2, 10]; therefore, the absence of adverse events should be interpreted with caution, as the present study was designed primarily to assess intraoperative PT measurement rather than clinical safety. Future studies should validate these findings in larger, multicenter cohorts. Further investigation into adaptive or soft tissue–compensated registration techniques may improve navigation reliability, particularly in patients with increased anterior soft tissue thickness. Additionally, incorporating functional pelvic assessments across multiple positions may provide a more comprehensive understanding of acetabular orientation during daily activities.

## CONCLUSIONS

The handheld imageless navigation device demonstrated moderate agreement with intraoperative radiographic and CT‐based planning measurements for assessing supine sagittal PT during primary DAA‐THA. Measurement errors were greater in patients with increased SPFT. These findings support the potential intraoperative utility of imageless navigation while highlighting the influence of anterior soft tissue thickness on measurement reliability. Therefore, it may be beneficial to utilize image‐based navigation for such patients, especially if they have additional risk factors for increased instability such as lumbar fusion; however, future studies are needed to address these gaps.

## AUTHOR CONTRIBUTIONS

Roham Borazjani, Danielle DeMoes and Carmelo Burgio drafted the manuscript. Tristan Jones, Stefan Kreuzer and Roham Borazjani generated the concept of the study. The acquisition of data and analysis were done by Danielle DeMoes, Tristan Jones and Roham Borazjani. Stefan Kreuzer, Anthony Gualtieri and Jonathan Vigdorchik provided supervision and guidance throughout the research process. The design of the study and the interpretation of data were all done by Roham Borazjani, Tristan Jones and Anthony Gualtieri. All authors revised the final draft critically for important intellectual content and approved the version to be submitted. All of the authors agreed to be accountable for all aspects of the work to ensure that questions related to the accuracy or integrity of any part of the work are appropriately investigated and resolved.

## CONFLICT OF INTEREST STATEMENT

Stefan Kreuzer reports receiving Royalties from Corin Group LLC and Stryker, serving as a paid speaker and consultant for Corin Group LLC, Naviswiss and Restor 3D, holding stock or stock options with Corin Group LLC, Naviswiss and Restor 3D. He is an unpaid consultant for Medtrade and CeramTec and is a board member for ISTA and PAS committee. Jonathan Vigdorchik reports receiving Royalties from Corin Group LLC, Depuy and Zimmer Biomet. He is on the Editorial Board at *Bone and Joint Journal* and is a Board member for the American Association of Hip and Knee Surgeons (AAHKS). The remaining authors declare no conflicts of interest.

## ETHICS STATEMENT

We used data from our registry. The registry was approved by the institutional review board (WCG IRB: 20203266). Written informed consent was obtained from patients to participate in our Surgical Joint Registry.

## Supporting information

Supporting File

## Data Availability

The data that support the findings of this study are available from the corresponding author upon reasonable request.

## References

[1] Abdel MP , von Roth P , Jennings MT , Hanssen AD , Pagnano MW . What safe zone? The vast majority of dislocated THAs are within the Lewinnek safe zone for acetabular component position. Clin Orthop Relat Res. 2016;474:386–391. 26150264 10.1007/s11999-015-4432-5 PMC4709312

[2] Ahmad SS , Hoos L , Perka C , Stöckle U , Braun KF , Konrads C . Follow‐up definitions in clinical orthopaedic research: a systematic review. Bone Jt Open. 2021;2:344–350. 34044582 10.1302/2633-1462.25.BJO-2021-0007.R1 PMC8168549

[3] Ashkenazi I , Thomas J , Lawrence KW , Meftah M , Rozell JC , Schwarzkopf R . The impact of obesity on total hip arthroplasty outcomes when performed by high‐volume surgeons—a propensity matched analysis from a high‐volume urban center. J Arthroplasty. 2024;39:1412–1418. 38428691 10.1016/j.arth.2024.02.066

[4] Borazjani R , DeMoes D , Hoveidaei AH , Kreuzer S . The impact of obesity on functional outcomes in navigation‐assisted total hip arthroplasty. Arch Orthop Trauma Surg. 2025;145:277. 40299074 10.1007/s00402-025-05889-7

[5] Dorr LD , Callaghan JJ . Death of the Lewinnek “safe zone”. J Arthroplasty. 2019;34:1–2. 30527340 10.1016/j.arth.2018.10.035

[6] Eilander W , Harris SJ , Henkus HE , Cobb JP , Hogervorst T . Functional acetabular component position with supine total hip replacement. Bone Joint J. 2013;95–B:1326–1331. 10.1302/0301-620X.95B10.31446 24078527

[7] von Elm E , Altman DG , Egger M , Pocock SJ , Gøtzsche PC , Vandenbroucke JP . The Strengthening the Reporting of OBservational studies in Epidemiology (STROBE) statement: guidelines for reporting observational studies. Int J Surg. 2014;12:1495–1499.25046131

[8] Frandsen JJ , Rainey JP , Kahn TL , Blackburn BE , Pelt CE , Anderson LA , et al. A novel method to calculate functional pelvic tilt using a standing anteroposterior pelvis radiograph. Arthroplast Today. 2023;21:101145. 37274836 10.1016/j.artd.2023.101145 PMC10238463

[9] General Assembly of the World Medical Association . World Medical Association declaration of Helsinki: Ethical principles for medical research involving human subjects. JAMA. 2013;310:2191–2194. 24141714 10.1001/jama.2013.281053

[10] Girardot G , Guy S , Ramos‐Pascual S , Dubreuil S , Saffarini M , Bonin N . No significant differences in 60‐day postoperative complication rates between conventional and shortened stems. J Exp Orthop. 2023;10:149–161. 38153605 10.1186/s40634-023-00696-8 PMC10754806

[11] Hasart O , Perka C , Christian K , Asbach P , Janz V , Wassilew GI . Influence of body mass index and thickness of soft tissue on accuracy of ultrasound and pointer based registration in navigation of cup in hip arthroplasty. Technol Health Care. 2010;18:341–351. 21209483 10.3233/THC-2010-0601

[12] Hasegawa M , Naito Y , Tone S , Sudo A . Accuracy of a novel accelerometer‐based navigation (Naviswiss) for total hip arthroplasty in the supine position. BMC Musculoskelet Disord. 2022;23:537. 35658945 10.1186/s12891-022-05495-3 PMC9166425

[13] Higa M , Tanino H , Ito H , Banks SA . Soft‐tissue tension during total hip arthroplasty measured in four patients and predicted using a musculoskeletal model. J Exp Orthop. 2023;10:130–139. 38051361 10.1186/s40634-023-00689-7 PMC10697917

[14] Hohmann E , Bryant A , Tetsworth K . Anterior pelvic soft tissue thickness influences acetabular cup positioning with imageless navigation. J Arthroplasty. 2012;27:945–952. 22036932 10.1016/j.arth.2011.09.017

[15] Imai N , Ito T , Suda K , Miyasaka D , Endo N . Pelvic flexion measurement from lateral projection radiographs is clinically reliable hip. Clin Orthop Relat Res. 2013;471:1271–1276. 23283671 10.1007/s11999-012-2700-1 PMC3586023

[16] Imai N , Suzuki H , Nozaki A , Hirano Y , Endo N . Correlation of tilt of the anterior pelvic plane angle with anatomical pelvic tilt and morphological configuration of the acetabulum in patients with developmental dysplasia of the hip: a cross‐sectional study. J Orthop Surg Res. 2019;14:323. 31623641 10.1186/s13018-019-1382-8 PMC6798456

[17] Kamenaga T , Hayashi S , Hashimoto S , Matsumoto T , Takayama K , Fujishiro T , et al. Accuracy of cup orientation and learning curve of the accelerometer‐based portable navigation system for total hip arthroplasty in the supine position. J Orthop Surg. 2019;27:1–6. 10.1177/2309499019848871 31104563

[18] Kromka JJ , Zuke WA , Granger CJ , Clohisy JC , Barrack RL . The CCJR® Charles A. Engh, Sr, MD. Excellence in Hip Research Award: pelvic tilt and cup position change significantly in most young patients 10 years after hip arthroplasty. J Arthroplasty. 2025;40:S25–S29. 10.1016/j.arth.2025.03.055 40147781

[19] Lembeck B , Mueller O , Reize P , Wuelker N . Pelvic tilt makes acetabular cup navigation inaccurate. Acta Orthop. 2005;76:517–523. 16195068 10.1080/17453670510041501

[20] Lewinnek GE , Lewis JL , Tarr R , Compere CL , Zimmerman JR . Dislocations after total hip‐replacement arthroplasties. J Bone Joint Surg Am. 1978;60:217–220. 641088

[21] Machida S , Tsutsumi M , Utsunomiya H , Ibara T , Kudo S . Characteristics of gait pelvic jerk in individuals with femoroacetabular impingement syndrome. J Exp Orthop. 2025;12:e70373. 40708883 10.1002/jeo2.70373 PMC12287974

[22] McCarthy TF , Alipit V , Nevelos J , Elmallah RK , Mont MA . Acetabular cup anteversion and inclination in hip range of motion to impingement. J Arthroplasty. 2016;31:264–268. 10.1016/j.arth.2016.01.067 27067753

[23] Novikov D , Mercuri JJ , Schwarzkopf R , Long WJ , Bosco JA , Vigdorchik JM . Can some early revision total hip arthroplasties be avoided? Bone Joint J. 2019;101–B:97–103. 10.1302/0301-620X.101B6.BJJ-2018-1448.R1 31146556

[24] Okamoto Y , Wakama H , Saika T , Tani K , Otsuki S . Association of spinopelvic mobility and osteosarcopenia with total hip arthroplasty outcomes. J Exp Orthop. 2025;12:e70395. 40766803 10.1002/jeo2.70395 PMC12322688

[25] Ong J , Ong CB , Grubel J , Chiu YF , Lee GC , Gonzalez Della Valle A . Body morphometry did not affect the accuracy of a second‐generation, miniature imageless navigation system for total hip arthroplasty (THA) using a posterior approach. J Clin Orthop Trauma. 2024;51:102404. 38638118 10.1016/j.jcot.2024.102404 PMC11021363

[26] Pierrepont J , Hawdon G , Miles BP , O'Connor B , Baré J , Walter LR , et al. Variation in functional pelvic tilt in patients undergoing total hip arthroplasty. Bone Joint J. 2017;99–B:184–191. 10.1302/0301-620X.99B2.BJJ-2016-0098.R1 28148659

[27] Roettges PS , Hannallah JR , Smith JL , Ruth JT . Predictability of pelvic tilt during total hip arthroplasty using a traction table. J Arthroplasty. 2018;33:2556–2559. 29656970 10.1016/j.arth.2018.03.018

[28] Scholes CJ , Fatima M , Schwagli T , Liu D . Imageless navigation system (Naviswiss) provides accurate component position in total hip arthroplasty with lateral decubitus position for end‐stage hip osteoarthritis: a prospective cohort study with CT‐validation. Arthroplasty. 2024;6. 10.1186/s42836-023-00224-0 PMC10773062 38191491

[29] Slotkin EM , Coxe F , Jones T , Morton T , Kreutzer S , Della‐Valle A . A handheld, portable image‐based system may outperform computer navigation or robotic platforms in providing accurate acetabular component positioning. Arthroplast Today. 2024;30:101511. 39959380 10.1016/j.artd.2024.101511 PMC11827077

[30] Stephens A , Munir S , Shah S , Walter WL . The kinematic relationship between sitting and standing posture and pelvic inclination and its significance to cup positioning in total hip arthroplasty. Int Orthop. 2015;39:383–388. 25132150 10.1007/s00264-014-2491-y

[31] Suzuki H , Imai N , Hirano Y , Endo N . Accuracy of acetabular cup implantation, as a function of body mass index and soft‐tissue thickness, with a mechanical intraoperative support device: a retrospective observational study. Acta Med Okayama. 2021;75:575–583. 34703040 10.18926/AMO/62770

[32] Takada R , Jinno T , Miyatake K , Hirao M , Yoshii T , Okawa A . Portable imageless navigation system and surgeon's estimate for accurate evaluation of acetabular cup orientation during total hip arthroplasty in supine position. Eur J Orthop Surg Traumatol. 2020;30:707–712. 31925538 10.1007/s00590-020-02625-2

[33] Yang G , Li Y , Zhang H . The influence of pelvic tilt on the anteversion angle of the acetabular prosthesis. Orthop Surg. 2019;11:762–769. 31663281 10.1111/os.12543 PMC6819173

[34] Yang Y , Zhao Z , Wang Y , Gao Y , Sun H , Liu W . Impact of wound complications in obese versus non‐obese patients undergoing total hip arthroplasty: a meta‐analysis. Int Wound J. 2023;20:4200–4207. 37518969 10.1111/iwj.14318 PMC10681413

