# Supplementary material for: Agreement of imageless navigation‐derived pelvic tilt measurements with radiographic and CT‐based measurements in direct anterior total hip arthroplasty: A prospective single‐center study
Source: J Exp Orthop. 2026 Jul 7;13(3):e70842. doi: 10.1002/jeo2.70842 (PMC13340135; doi:10.1002/jeo2.70842)
Supplement: Supplementary file 1 — Supporting File [file JEO2-13-e70842-s001.docx]

| **Table S1. Preoperative Functional outcome scores** | | |
| --- | --- | --- |
| **Preoperative HOOS** | **Mean ± SD** | **Range** |
| Symptom | 44.9 ± 18.6 | 0-75 |
| Pain | 42.7 ± 16.7 | 8-78 |
| ADL | 49.4 ± 19.1 | 18-96 |
| Sport | 30.3 ± 21.6 | 0-94 |
| QoL | 21.9 ± 20.5 | 0-69 |
| HOOSJR | 47.6 ± 15.2 | 15.6-80.6 |
| **Preoperative UCLA*** | 5.4 ± 2.6 | 2-10 |
| HOOS(JR)= Hip Disability and Osteoarthritis Outcome Score (for Joint Replacement), ADL= Activity of Daily Living, QoL= Quality of Life. | | |
